# Uncertainties in Dimensional Measurements Made at Nonstandard Temperatures

**DOI:** 10.6028/jres.099.004

**Published:** 1994

**Authors:** Dennis A. Swyt

**Affiliations:** National Institute of Standards and Technology, Gaithersburg. MD 20899-0001

**Keywords:** dimensional measurement, dimensional tolerances, length metrology, measurement uncertainty, reference temperature, thermal expansion

## Abstract

This report examines the effects of uncertainties in temperature and coefficient of thermal expansion on the expanded uncertainty of length dimensional measurements made away from the international standard reference temperature of 20 °C for artifact standards and workpieces of various materials. Specific cases examined deal with: 1) uncertainties of thermal-expansion coefficients associated with values given in engineering references, standard reference data, standard reference materials and direct measurements; and 2) uncertainties of part temperature measurements associated with realizing the International Temperature Scale of 1590 (ITS-90) and determining part temperatures relative to ITS-90 with the principal types of thermometry and achievable levels of temperature control.

## 1. Introduction

Material objects—whether complex-geometry parts designed to fit into assemblies or simple-geometry artifacts designed to be calibrated as standards of length — have dimensions which vary with temperature. The size of the variation depends upon the specific material. For example, for aluminum, steel, and silicon, typical coefficients of thermal expansion are respectively, in units of parts per million per degree Celsius, 23.1 ppm/°C, 11.5 ppm/°C, and 2.6 ppm/°C.

Because of the effects of thermal expansion, by national and international agreements length-based dimensions—including those specified, for example, on engineering drawings — are defined to be those which exist at a standard reference temperature of 20 °C [1,2].

**Fig. 1:**
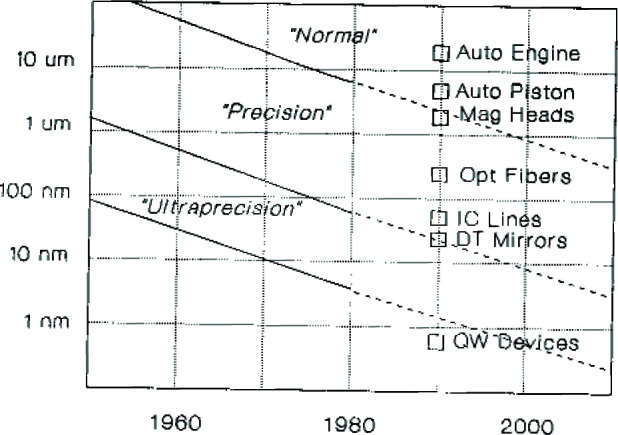
Trends and examples of state-of-art in dimension tolernces of manufactured parts in normal, precision, and ultrprecision regimes.

Figure 1 illustrates one of two recent developments which have made the issue of thermal-expansion effects in part metrology a matter of increased concern. The figure shows the on-going trend in the manufacture of discrete-part products to increasingly tighter dimensional tolerances in state-of-the-art manufactured goods from aircraft and automobiles to computers and electronics [3]. According to this trend, such tolerances have been decreasing in size by a factor of approximately three every ten years, so that there are today, for example, automobile pistons with tolerances of 6 µm–7 µm and quantum-well electronic devices with tolerances of 0.5 nm [4].

The second development is a proposal to the International Organization for Standardization, subsequently unadopted but of technical import, to change the international standard reference temperature for dimensional measurements from 20 °C to 23 °C [5]. Since referring measurements to a standard temperature serves to reduce actual variations in dimensions of parts due to thermal-expansion effects as well as uncertainty in measurements, a shift in reference temperature can increase each, that is, both variations and uncertainties.

This paper looks at possible errors and likely uncertainties in dimensional measurements due to thermal-expansion effects where those measurements are made away from the reference temperature, either the specific interval of 3 °C due to a change to the proposed 23 °C or an arbitrary interval due, for example, to the settling of a temperature control system at other than the standard reference temperature.

## 2. Uncertainties Due to Thermal Expansion

Contributions to uncertainty in measurements of length-based dimensions due to measurements made at nonstandard temperatures are a function of the length of the object being measured, its temperature, its coefficient of thermal expansion, and the uncertainties in each of these quantities.

The coefficient of linear thermal expansion (CTE) of a material, *α*, is defined to be

$$\alpha \left(T\right)=\frac{dL/L}{dT},$$ (1)

where d*L/L* is the fractional change in a characteristic linear dimension and d*T* is the change in temperature. For a sample with length *L*_0_ at temperature *T*_0_, the length *L* at temperature *T* is found by integration to be

$$L=L_{0}\exp\left[\int_{T_{0}}^{T}\alpha \left(T\right)dT\right].$$ (2)

If *α* (*T*) is assumed to vary only slightly over the temperature range *T − T*_0_, it may be replaced by an average value *α* and Eq. (2) becomes

$$L=L_{0}\;\exp\;\left[\alpha \left(T-T_{0}\right)\right].$$ (3)

For typical materials and for changes of temperatures from room temperature to their melting points, Eq. (3) is approximated to within less than 1% by

$$L=L_{0}\;\left[1+\alpha \left(T-T_{0}\right)\right].$$ (4)

Equation (4) is the standard expression used to correct dimensional measurements made at a uniform temperature other than the one desired.

## 3. Uncertainties and Error Relative to Tolerances

This report will use two different methods for examining the effects of thermal expansion relative to tolerances of measurements made at nonstandard temperatures. The first method follows the recommended practice of an international standards body and deals with propagated uncertainties. The second method follows the recommended practice of a national standards body and deals with estimated maximum error. Each method compares resulting uncertainties to a tolerance, that is, to a specified limit of permissible error.

### 3.1 Thermal Uncertainty Index (TUI)

The first method—which is based upon the approach recommended by the International Committee for Weights and Measures (CIPM), which is the basis of a guideline published by the International Organization for Standardization, and which has been adapted as NIST policy—uses root-sum-of-squares (RSS) propagation of uncertainty [6]. In this approach, the combined standard uncertainty associated with the correction for thermal expansion given by Eq. (4) is the positive square root of the estimated variance *u*_c_^2^ given by

$$u_{c}=\sqrt{{\left(\frac{\partial L}{\partial T}\right)}^{2}{u_{T}}^{2}+{\left(\frac{\partial L}{\partial \alpha }\right)}^{2}{u_{\alpha }}^{2},}$$ (5)

where there is assumed to be no correlation between the variations in temperature and the variations in the coefficients of thermal expansion. Following the CIPM approach, in this first method results are expressed as an expanded uncertainty:

$$U=k\cdot u_{c},$$ (6)

with *U* determined from a coverage factor *k and* the combined standard uncertainty *u*_c_, the estimated standard deviation given by Eq. (6). To be consistent with current international practice, the value of *k* used by NIST for calculating *U* is, by convention, *k* = 2 [7]. Hence, with partial derivatives from Eq. (4), substitution of Eq. (5), and $u_{T_{0}}=0$, Eq. (6) becomes

$$U=2\cdot u_{c}=2\sqrt{{\left(\alpha L{\;}_{0}u_{T}\right)}^{2}+{\left(u_{\alpha }L_{0}\left(T-T_{0}\right)\right)}^{2}}.$$ (7)

In parallel with the method to be described in the next section, this paper defines a ratio of expanded uncertainty to tolerance, that is, the limit of permissible error, called the Thermal Uncertainty Index (*TUI*):

TUI=(U/τ)×100%, (8)

where *U* is the expanded uncertainty defined by Eq. (7) and *τ* is an engineering tolerance specific to a given situation.

### 3.2 Thermal Error Index (TEI)

The second method, based on the approach recommended by the American National Standard Institute (ANSI) in its standards dealing with environmental conditions for dimensional measurements, involves linear addition of absolute values to estimated limits of error [2]. In this approach, the estimated worst-case limit of error *c*_e_ associated with the correction for thermal expansion given by Eq. (4) is

$$e_{c}=\left|\frac{\partial L}{\partial T}\right|e_{T}+\left|\frac{\partial L}{\partial \alpha }\right|e_{\alpha },$$ (9)

which, with partial derivatives from Eq. (4), becomes

$$e_{c}=\left|\alpha L_{0}\right|e_{T}+\left|L_{0}\left(T-T_{0}\right)\right|e_{\alpha },$$ (10)

where *e_τ_* and *e_α_* are worst-case errors in temperature and thermal-expansion coefficients and the terms proportional to each are the errors in the correction for thermal expansion due respectively to nominal differential expansion and the temperature variation.

In the ANSI standard which specifies the temperature conditions for dimensional measurements, Thermal Error Index (*TEI*) is defined and represented formally by:

TEI=[(TVE+UNDE)/WT]×100%⩽50%, (11)

where *TEI* is the thermal error index, *UNDE* is the stated uncertainty (no further specification) of nominal differential expansion times the temperature difference, *TVE* is a temperature variation error (defined by a maximum range of temperature drift), and *WT* is the working tolerance for a specific test. According to ANSI-standard procedures for evaluating the performance of dimensional measuring machines, the *TEI* should be less than 50% [8].

The parallelism of the two terms of the Thermal Error Index given by Eq. (11) with those of the variational form of thermal-expansion errors on length given by Eq. (9) suggests that a useful basis for estimating the significance of thermal-expansion effects in dimensional measurements in a specific situation is to determine whether the ANSI-specified condition on *TEI* is met, that is, whether the worst-case limit of error defined by Eq. (10) meets the following condition:

$$e_{e}/\tau \;⩽\;1/2,$$ (12)

where *WT*, the symbol for the working tolerance used in the standard, has been replaced by *τ*, the symbol for the specified tolerance introduced in the definition of Thermal Uncertainty Index defined in Eq. (8).

### 3.3 Interpretation of Statements of Accuracy, Uncertainty, and Error

This report follows the NIST policy on statements of uncertainty associated with measurement results which gives procedures for combining various statements of accuracy, uncertainty and limits of error from other sources, including published measurement data, manufacturer’s specifications, data in calibration and other reports, and reference-data handbooks [9].

Throughout this report, unless otherwise noted, unqualified statements of accuracy, uncertainty and limits of error that are taken from other sources are indicated as “stated uncertainty” (designated in Tables by the symbol *Δ*) and discussed as such, but, when combined, are converted to the standard-uncertainty representation by assuming a uniform or rectangular probability distribution with

$$U=2\;u=\frac{2}{\sqrt{3}}a=1.155\;a,$$ (13)

where *a* is the stated accuracy, uncertainty or estimated limit of error in the reported source and the half width of the assumed distribution. Thus a value given in some source as *“Y±X%”* is quoted here as a stated uncertainty of *X* but when combined to give an expanded uncertainty is represented as *“Y±*1.155 × *X%.”* Note for comparison that this method of conversion to an expanded uncertainty yields a result which is within 15% of both the unqualified original statement and a value reported at the 95% level of confidence, which is converted to the 2*σ* expanded uncertainty by multiplication by 2/1.96, but is that much outside the assumed uniform distribution and is, therefore, non-physical. Note, however, that since both are so converted, the ratio of the uncertainty to a tolerance is the same whether in the stated or expanded forms.

## 4. Uncertainties Due to Variations in Coefficient *α*

An uncertainty in measurement results from uncertainty in the particular value of the CTE, *α*, used to calculate a part’s dimension at the reference temperature when measurements are made at another temperature. The uncertainty in the nominal CTE, while seldom considered in conventional dimensional metrology, has long been recognized as important for large parts (large *αL*_0_) and for large temperature extrapolations (large *T* − *T*_0_) [2,10]. Due to the trends which have made micrometer and nanometer tolerances more commonplace, errors and uncertainties due to thermal-expansion effects are now an important consideration for part sizes and temperature extrapolations not previously considered large.

### 4.1 Range of Reference Values of *α*

Table 1 shows the variety of values of CTEs of some metrologically important materials that can be found in references including handbooks for engineers, machinists, and material scientists. Among the materials are: the elements aluminum, iron, and silicon; specific alloys such as Al 6061 and stainless steel 304; general alloys such as cast iron and carbon steel; common Pyrex^1^ (a borosilicate glass) and low-expansion materials, including vitreous silica (fused polycrystalline quartz) and Zerodur (a mixture of crystalline and polycrystalline quartz) (11–17). Inspection of Table 1 shows the problem of determining a value of CTE for a specific object by looking up a value for a material, namely the variety of values likely to be encountered.

Variations among the values for the various materials from the references shown in Table 1 include, for example, 4.5 ppm/°C or 35% of the mid-range value for carbon steel, 7.0 ppm/°C or 50% of the mid-range value for the stainless steel (which includes CTEs for SS-301 and others from a reference which gives CTEs only for generic types of SS), and 11.2 ppm/°C or 75% of the mid-range for cast-iron.

Table 2 illustrates some likely causes for such variations in tabulated values of CTEs, with the 35% range of the extremes from the mid-value CTE encountered for carbon steel taken as an example. As with other materials, these causes of variations are differences in chemical composition, the physical processing to which the specific sample has been subjected, and the value or range of temperatures for which the coefficient is specified.

The first likely cause of differences in reported values of CTEs for nominally the same material is differences in chemical composition. In general, the name carbon steel encompasses a range of carbon concentration from a few tenths of one percent to nearly 1.5% and includes various small amounts of other elements such as Mn, P, S, Si, Cr, Ni, or Mo, with the values of CTE of annealed samples of carbon steels reported by one source ranging from 11.1 ppm/°C to 12.6 ppm/°C depending on composition [14].

The second likely cause of differences in reported values of CTEs for nominally the same material is differences in microstructure associated with the physical processing to which the sample of material has been subjected. These processes include combinations of mechanical working and heat treatment, such as hot rolling, cold rolling, drawing, casting and annealing. For example, the range of variation of the CTE of steel has been reported to be ±2% (0.2 ppm/°C) among samples cut from different locations in a large piece of steel that has been fully annealed, ±3% (0.3 ppm/°C) among many heats of nominally the same chemical content, ±5% (0.5 ppm/°C) between hot and cold rolling, and ±10% (1.1 ppm/°C) among several heat treatments [18]. For the carbon steel (AISI 52100) of gage blocks, the annealed and hardened states of the material have reported CTEs (20 °C to 100 °C of 11.9 ppm/°C and 12.6 ppm/°C, respectively [15].

In the case of Invar, Table 1 shows a range of values of CTE from 0.13 to 2.0 ppm/°C for various types of mechanical working and heat treating. Such processing can increase or decrease CTEs and can yield positive, negative or zero values, each of which can vary with time. As indicated by Table 3, annealing of Invar can increase the CTE and quenching can decrease it. Cold working after quenching can reportedly produce a negative coefficient, with very low CTEs usually reverting with time to the normal value for the material [15].

The third likely cause of differences in reported values of CTEs for nominally the same material are differences in the values or range of temperatures for which the CTEs are given. Among the sources cited here the most typical situation is an average value for a range of temperature from 20 °C up to 100°C or as much as 1000 °C. That such average values can be significantly different than the 20 °C standard-temperature value is shown by Table 4, which compares with its 20 °C value the mean CTE for the range 20 °C to 107 °C and also shows the temperature derivative of the CTE at 20 °C in both a ppm/(°C)^2^ and %/*°C* form [16,17], Note that for some materials the difference between the CTE at 20 °C and an average value, such as that for the range 20°C–107°C shown, can be substantial, including 1 ppm/°C (5%) for aluminum and its alloys, 0.5 ppm/°C (20%) for silicon, and 0.43 ppm/°C (300%) for Invar.

A further consideration in assigning a value of CTE to a particular object is whether the material of the object is homogenous. An obvious situation is that of a compound object, that is, an assembly consisting of materials with different coefficients. One example of such is a commercial bait-plate for performance evaluation of coordinate measuring machines, which consists of ceramic balls mounted in a steel plate [19]. Less obvious is the situation of case-hardened parts, where the surface to some depth has a different CTE than that of the interior. Due to such inhomogeneities, measured values of CTE for steel gage blocks have been observed to be length-dependent, ranging from an asymptotic 12.0 ppm/°C for lengths less than 50 mm to an asymptotic 10.6 ppm/°C for lengths greater than 500 mm, with a value of 11.5 ppm/°C for lengths near 100 mm [20].

### 4.2 Uncertainty in Specific Values of *α*

Given that the CTE of an object depends upon it; homogeneity, chemical composition, history of thermal-mechanical processing (such as heat treatment cold working, and hardening), and temperature, a basis for estimating the degree to which even well-characterized values of CTE are known is given by Table 5, which shows the slated uncertainties in CTEs for some calibration artifacts, standard reference data and standard reference materials.

As indicated in the first row of Table 5, the American National Standard ANSI/ASME B89.1.2 for gage blocks specifies that the CTEs of gage blocks conforming to the standard are stated to be “accurate to within ± 10% of value stated for the blocks between 15 °C and 30 °C” [21]. The parallel international standard specifies that the CTE of steel gage blocks in the temperature range 10 °C and 30 °C be within the limits (11.5 ± 1.0) ppm/°C, an 8.7% tolerance [22].

Shown in the second row of Table 5 are the stated values of uncertainty specified with standard-reference-data values of CTE for materials covering a wide range of values [16]. As indicated by Table 5, typical reported uncertainties for what are averages over a number of well-annealed samples of specific-composition alloys are 5% and 7%.

In the third row of Table 5 are the stated uncertainties assigned to the values of CTEs of standard reference materials produced and sold as standards of thermal expansion for use in calibrating dilatometers [23]. As indicated, the stated uncertainty associated with each of these specific well-annealed samples of specific-composition reference materials is ±0.03 ppm/°C, which for materials such as steels with coefficients of the order of 10 ppm/°C corresponds to approximately 0.3%.

Finally, in the fourth row of Table 5 are the stated uncertainties of recent dilatometer measurements by a national standards laboratory on a range of materials, including, for example, one of the standard reference materials shown in the third row [24]. As indicated, the reported uncertainties for each of these materials vary from a high of 0.086 down to a low of 0.006 ppm/°C. Representative of the stated uncertainties in the CTEs of these standard reference materials is the 0.36% value for the materials other than the zero-expansion glass-ceramic.

Taken together, Tables 1, 2, and 5 provide a basis for some generalizations about the expanded uncertainties of values of CTEs: First, with no further information about composition or history, the expanded uncertainty of the CTE for materials simply described as carbon steel, stainless steel or cast iron can be from 5 ppm/°C to greater than 10 ppm/°C (as indicated by Table 1 which includes ranges of reported values of 4.5 ppm/°C or 35% of the mid-range value for carbon steel, 7.0 ppm/°C or 50% of the mid-range value for stainless steel 304, and 11.2 ppm/°C or 75% of the mid-range for cast-iron).

Second, knowing only that a material is gage-quality carbon steel, tungsten carbide or chromium carbide, the expanded uncertainty of the material’s CTE is likely to be of the order of 10% or 1 ppm/°C.

Third, with information about chemical composition, the expanded uncertainty in the tabulated values of CTEs of a variety of standard-composition substances including metals, alloys and non-metallic materials are usually of the order of 6% to 9%. (With this generalization, one should keep in mind that the standard reference data are usually for well-annealed specimens of a class of materials and sometimes includes an average over a range of compositions.)

Lastly, with direct measurements of CTEs obtained by dilatometry on particular specimens of materials with coefficients in the range of, say, 3 ppm/°C (such as silicon) to 23 ppm/°C (such as aluminum and its alloys), the expanded uncertainties in CTE are of the order of 0.3%.

## 5. Uncertainty in Temperature

Uncertainty in the measurement of the length of a part also results from the uncertainty in the value of the temperature of the part, because the temperature must be measured and used to calculate the part dimension at the reference temperature.

### 5.1 Sensor-Limited Uncertainty in Temperature Measurement

Table 6 shows representative limiting uncertainties, stated (*Δ_τ_*) and expanded (*U_τ_*), associated with the use of the major types of NIST-calibrated temperature sensor systems for the determination of an object’s temperature and, for reference, the absolute limit of temperature measurement at 20 °C This limit is the 0.0002 °C expanded uncertainty of a primary calibration of a SPRT, which is also the uncertainty with which the melting point of gallium, a defining point on the International Temperature Scale, can be realized [25].

In order of decreasing values, the stated (and expanded) uncertainties are: 1) 0.1 °C (0.12 °C) for a Type-T thermocouple with a reference junction in an ice bath and read-out with a digital voltmeter [26]; 2) 0.03 °C (0.035 °C) for a visually-read mercury-in-glass thermometer [26]; 3) 0.01 °C (0.012 °C) for well-selected glass bead thermistors [27]; 4) 0.002 °C (0.0023 °C) for Type-T thermocouples referenced directly against a standard platinum resistance thermometer (SPRT) in a temperature-controlled 20 °C cell [28]; and 5) 0.001 °C (0.002 °C) for one SPRT as sensor referenced against a second in a 20 °C cell [25].

### 5.2 Object Temperature Measurement

**Fig. 2:**
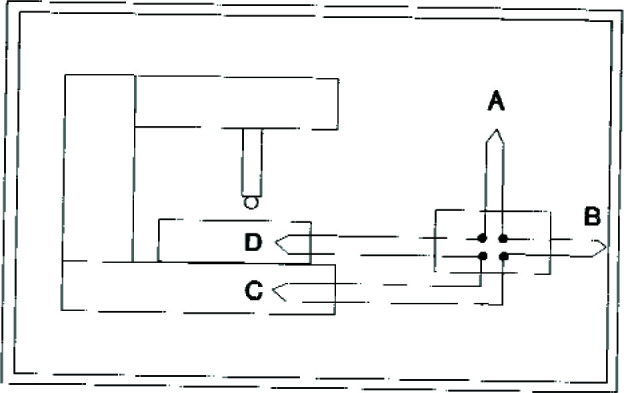
Schematic representation of alternative locations of temperature monitors: (A) air surrounding object; (B) enclosure walls; (C) machine; (D) object of measurement itself.

Figure 2 shows schematically the types of locations at which temperature measurements are made: (A) in the air (or liquid) medium surrounding the object or part the temperature of which is to be determined; (B) on the walls of the temperature-control enclosure surrounding the measuring machine; (C) on the measuring machine; or (D) on the object itself.

Because combinations of radiation, convection, and conduction within this overall system can produce differential heating or cooling, the temperature of the part as a whole is not necessarily the same as that of any these points of measurement, including a single point on the object. Uncertainty also results from nonuniformity of the temperature distribution over the part, or nonequilibrium of the part with the environment at which temperature is measured.

### 5.3 State-of-the-Art Temperature Facilities

Table 7 shows, for state-of-the-art measuring and manufacturing systems, the stated temperature “stability” of each (taken to be the temporal variation about a mean temperature) and reported temperature “accuracy” (taken to be the stated uncertainty in that mean temperature). In each case, stated stabilities and accuracies are each treated as otherwise-unspecified single-component uncertainties obtained from quantities with uniform distribution and converted to expanded uncertainties by multiplication by 1.155.

In the order of decreasing expanded uncertainty, these systems include: (1) conventional metrology facilities with temperatures controlled to 0.12 °C; (2) two commercial laser-interferometer microelectronics mask measurement systems with stabilities of 0.058 °C and 0.012 °C, respectively [29,30]; (3) Physicalish-Technische-Bundesanhalt’s special metrology facility controlled to 0.012 °C [31]; (4) Lawrence-Livermore’s Large Optics Diamond Turning system with a measured stability of its surrounding air environment of 0.001 °C and an expanded uncertainty of 0.012 °C [32]; and (5) NIST’s Linescale Interferometer System with a temperature measurement expanded uncertainty of 0.0023 °C [28].

## 6. Thermal-Expansion Analyses of State-of-the-Art Engineering Measurement Systems

Table 8 shows reported results of analyses of thermal expansion effects in three state-of-the-art engineering measurement systems. The systems are: 1) a specialized measuring machine for inspecting the mating features of the solid rocket motor of the U.S. Space Shuttle; 2) a commercial high-accuracy coordinate measuring machine used, for example, in automobile manufacturing; and 3) a specialized metrology system required for measurement of new-generation x-ray lithography masks. Based on stated uncertainties in thermal expansion (*Δ_a_*) and temperature (*δ_t_*), the stated uncertainties are represented in incremental length (40, fractional length (*Δ_L_/L*), and fractional tolerance (*Δ_L_*/*τ*) forms and compared with the ANSI-Standard Thermal Error Index (*TE1*).

### 6.1 Solid Rocket Motor Seal

In the second column of Table 8 are shown data and results of an analysis of the stated measurement uncertainties of a special-purpose profile measuring device developed for the U.S. space program to measure the absolute diameters of mating features of the redesigned joints of the Space Shuttle solid rocket motor subsequent to the failure which destroyed the Challenger [10]. The analysis deals with the case of an aluminum-arm measuring device calibrated at one temperature and used to measure the 3.65 m (144 in) diameter of a steel part at another temperature as much as 11.1 °C (20 °F) different. Machine and part temperatures are stated to be controlled to ±0.27°C (0.5 °F). With use of reference-table values of CTE of aluminum and steel, assumption of stated uncertainties in CTEs of ±5%, and linear addition of absolute values of probable errors, the result of the analysis is that the machine’s stated uncertainty is 95.3 μm (0.00375 in), representing 27 ppm of part size and 75% of the specified 127 μm (0.005 in) tolerance. The analysis also notes that with machine calibration and part measurement carried out under the improved temperature conditions of (20.0±0.36) °C [(68.0 ±0.2) °F] noted in Table 7 as ideal, the machine’s stated uncertainty improves to 17.6 μm (0.0021 in) which is 4.8 ppm and 14% of tolerance, that is, of maximum permissible error.

### 6.2 High-Accuracy Coordinate Measuring Machine

In the third column of Table 8 are shown data and results of the vendor’s analysis of the stated measurement uncertainty of a commercial coordinate measuring machine (CMM) of the type used, for example, in the aerospace and automobile industries [33]. The problem is to determine under what thermal-expansion conditions it can be determined that a CMM performs within its stated uncertainty:

$$U_{1}/\mu m=0.5+\left[L/\text{mm}\right]/1200$$ (14)

using a step gage with stated calibration uncertainty:

A/μm=0.05+[L/mm]/2000 (15)

where *U*_1_ is the single-axis linear uncertainty for CMMs stated in the form specified by the German industrial standard [34] and *A* is the vendor-stated calibration uncertainty of the step gage, and *L* is distance in mm.

The vendor’s analysis deals with the case of using a step gage one meter in length at a temperature chosen to be 21 °C controlled to ±0.1 °C under four conditions: step gage of either steel with a CTE of (11.5 ± 0.1) ppm/°C or Zerodur with a CTE of (0.00 ±0.05) ppm/°C and uncertainties combined either in absolute values or root-sum-of-squares. The reported result is that the machine can only be satisfactorily determined to perform to a stated uncertainty of 1.33 μm at one meter using the Zerodur gauge, When added in absolute values and root-sum-of-squares, the resulting uncertainty in measurements with the steel step gage comprise respectively 135% and 96% of the tolerance. With the Zerodur step gage, in each case they comprise less than 50%, the implication being that the use of a Zerodur gage more satisfactorily allows the machine’s performance to be judged to be within the manufacturer’s stated uncertainty.

### 6.3 X-Ray Lithography Photomask

In the fourth column of Table 8 are shown data and results of a national laboratory’s analysis of the stated uncertainty required to calibrate a reference dimensional standard for x-ray lithography photomasks [35]. The analysis deals with the case of a one-gigabit DRAM device and the reductions in uncertainties required at each successive level of the process; a critical dimension (CD) of 175 nm to 200 nm, with error of overlays (EOW) on wafers of CD/2.5, image placement accuracy (IPA) on masks of EOW/3, required industrial reference metrology accuracy (IRM) of IPA/4 and required national laboratory uncertainty of IRM/4, the resulting uncertainty required of the national laboratory is 1.25–1.75 nm, shown in Table 8 as a tolerance, i.e., permissible limit on measurement uncertainty, of 1.5 nm. Based on a reference-table value of CTE for silicon known to *±*3*%* [16], the analysis shows that measurements made at the 20 °C reference temperature to a state-of-the-art level of temperature control of 0.01 °C yield an expanded uncertainty of 1 nm, representing 0.02 ppm of positional accuracy on the 50 mm wafer and 67% of the tolerance on the stated calibration uncertainty.

For each of the three examples, Table 8 also gives calculated values of *TEI* and shows the following results. In the rocket-motor example, the worst-case uncertainties due to differential thermal-expansion effects of the measuring arm and part just meet the ANSI B89 standard condition of TEI/⩽50%. In the CMM example, while the test with the Zerodur step gauge meets that condition, that with the steel step gauge does not. In the x-ray mask example, for that condition to be met a uniform part temperature known to better than 0.01 °C is required.

## 7. Limiting Situations in Calibrations and Measurements

Based on the results of previous sections, Table 9 shows for various measurement situations the uncertainties in length measurements in terms of increments, fractions of the dimension measured and fractions of specified tolerances on the two bases described in Sec. 3. In the middle section of Table 9 are given stated uncertainties for CTE (*Δ_α_*) and temperature (*Δ_t_*) combined in absolute values according to Eq. (10) to provide a stated uncertainty in length (*Δ_L_*), and *TEI*, and comparison to a stated tolerance (*τ*) as in Eq. (11). In the lower section of Table 9 are given expanded uncertainti for *CTE* (*U_α_*) and temperature (*U_T_*) combined sum-of-squares to provide an expanded uncertair in length (*U_L_*), a *TUI*, and comparison to an ϵ panded-uncertainty tolerance (*τ*) as in Eq. 8.

### 7.1 Limit of Definition of Temperature

The second column in Table 9 shows that from materials having CTEs in the range of nominal values 2.5 ppm/°C to 25 ppm/°C (which includes materials from silicon through steel to aluminum), the current ±0.0001 °C standard uncertainty of the definition of temperature corresponds to a standard uncertainty in length ranging from 0.25 to 2.5 nm at 1 meter. Given that, by international and national standard, the length of an object is defined at a uniform temperature of 20°C and that uncertainty of temperature measurement is limite by that of the ITS-90 temperature scale at that reference temperature uncertainty, the second column of Table 9 shows that the corresponding expanded uncertainty in length measurement of 5×10^−10^ represents the current absolute limit for which a standards-defined length of the material indicated can be determined.

For reference, Table 10 shows the limiting value of relative expanded uncertainty of length measurements (*u_l_/l*) for material objects of low expansion materials imposed by ITS-90 compared to the expanded uncertainty in the wavelength of the iodine-stabilized helium-neon lase (δλ/λ =5.0×10^−11^ [36]) by which the SI unit of length in engineering measurements is practically defined. Table 10 also shows the value of CTE, 0.3 ppm/°C, at which the contributions to the uncertainty in a length measurement of the uncertainty in the ITS-90 temperature scale and of the reference wavelength are equal, that is, where

$$U_{L}/L=\alpha U_{\text{ITS}}={\left(U_{\lambda }/\lambda \right)}_{\text{HeNe}}.$$ (16)

As indicated in Table 10, for materials with CTEs greater than the 3.4 ppm/°C, which value is near that of silicon and Pyrex (borosilicate glass), a lower limit of the uncertainty of any measurement of the length of a material object is imposed by the uncertainty in temperature defined by ITS-90, while for materials with CTEs less than 3.4 ppm/°C (including Invar, fused silica, and Zerodur) a larger uncertainty is imposed by the uncertainty in the wavelength of the iodine-stabilized laser.

### 7.2 Limit of Realizing 20 °C in the Laboratory

The third column of Table 9 shows again for materials ranging from silicon to aluminum that the practical limit of realizing 20 °C, achievable with high- but not ultimate-performance equipment such as an $8\frac{1}{2}$-digit voltmeter and best laboratory practice, is about ±0.001 °C [25,37]. This temperature corresponds to an expanded uncertainty in length of 2,5 nm-25 nm at one meter. Thus, in terms of fractional length (*U_T_*/*L*), 3 parts in 10^9^ currently represents the lowest uncertainty with which a length of a material object can be determined.

### 7.3 Design Goal of M3

The fourth column of Table 9 shows the design parameters and performance goals for the Molecular Measuring Machine (M3), a laser-interferometer and STM-probe planar coordinate measuring machine being constructed at the National Institute of Standards and Technology [38]. With the SPRT-SPRT thermometry described in Table 6 in which one SPRT acts as sensor for monitoring the temperature of the part carrier and the other acts as the reference, the M3 goal is to be able to achieve for a silicon wafer of 70 mm diagonal a temperature which is uniform, stable and accurate to 0.001 °C, the current practical limit of realizing the reference temperature in a laboratory. For M3 that limit of measurement of part temperature corresponds for a silicon part to an expanded uncertainty in length of 0.22 nm at 70 mm (3.1 part in 10^9^) or 18% of the machine’s design goal for point-to-point position measurement.

### 7.4 Primary Calibration Laboratory

The fifth column of Table 9 shows operational parameters for the NIST Length Scale Interferometer, a primary-calibration facility the accuracy of which is checked through international round-robins aimed at assessing the capabilities of national laboratories in various industrialized nations to realize the definition of the meter [28]. For the steel meter-bar used in such intercomparisons, the stated 0.002 °C expanded uncertainty in part temperature and offsets of no more than 0.01 °C from the 20 °C reference temperature corresponds to 24 nm at 1 m, which is 2.4 parts in 10^8^ or 25% of a nominal expanded uncertainty of 0.1 μm at a meter.

### 7.5 Secondary Calibration Laboratory

The sixth column of Table 9 shows operational parameters of a hypothetical secondary calibration laboratory representative of current good practice among industrial and government metrology facilities. With CTEs known to the standard-reference-material level of ±0.03 ppm/°C of Table 9, temperature controlled to a state-of-the-art facility level of ±0.01 °C, and part temperature offsets from the reference temperature of no more than 0.1 °C, the thermal-expansion contribution to length measurement expanded uncertainty corresponds to 0.14 μm at 1 meter or 1.4 parts in 10^7^, which represents 14% of an expanded uncertainty of 1.25 μm at a meter representative of the uncertainties of today’s high-performance CMMs.

### 7.6 Tertiary-CaUbration/Industrial-Inspection Laboratory

The seventh column of Table 9 shows operational parameters of a tertiary calibration laboratory which can be representative of industrial part-inspection facilities. With CTEs assumed to be known to standard-reference-data values of ±5%, temperature controlled to a conventional metrology facility level of ±0,1 °C, and part temperature offsets from the reference temperature of no more than 1 °C, the thermal-expansion contribution to possible error corresponds to 1.8 μm at 1 m or 2 parts in 10^6^, which represents 15% of the 12 μm tolerances on transmission housings, clutch covers, and meter-size automobile engine blocks and cylinder heads measured by commercial CMMs [39].

### 7.7 Change in Reference Temperature

The eighth and ninth columns of Table 9 show two examples of the effects on possible error in industrially important applications cited in research and trade journal editorials concerning the recent unadopted ISO proposal to change the standard reference temperature for dimensional measurements from 20 °C to 23 °C [40,41].

The example shown in column 8 of Table 9 is that of a calibrated steel lead screw in a machine tool or measuring machine. The CTEs of even highly controlled products such as gage blocks or lead screws is said to be known to vary from lot to lot by 5%. Here it is shown that, with a standard still calibrated at 20 °C, this stated 5% uncertainty in the CTE coupled with the 3 °C temperature shift would give rise to a possible error contribution of 1.8 parts in 10^6^, which would add 1.8 μm/m or 5% to the 33.3 μm/m (0.0004 in/ft) value cited as the error of the steel lead screw of an accurate machine tool, an amount which is considered significant in terms of the machine’s intended limit of permissible error.

The example shown in the last column of Table 9 is that of a nominally 100 mm-diameter (4 in) aluminum engine piston. In this case, the dimension of the part measured at a “new” reference temperature of 23 °C has an expanded uncertainty relative to that of a part designed to be measured at the “old” reference temperature of 20 °C of 7 μm or nearly the entire 7.6 μm (0.0003 in) of the initial tolerance for piston-to-cylinder fit for many engines, an effect considered likely to be improperly compensated and a major potential problem in fit and function.

## 8. Conclusion

This paper has examined the effects on uncertainties in dimensional measurements due to uncertainties in the temperature and coefficients of thermal expansions for both physical-artifact standards and manufactured workpieces. The motivations for this examination are both the trends to tighter tolerances in discrete-part manufacturing which pose great challenges in dimensional metrology, and also the proposed change in the standard reference temperature at which such dimension are defined.

The paper’s principal conclusion is that, under common conditions of temperature measurement and knowledge of coefficients of thermal expansions of engineering materials, the contributions of thermal-expansion effects to measurement uncertainty are frequently large, sometimes dominant and occasionally overwhelming factors relative to tolerances specified in precision-tolerance manu facturing.

This paper also shows that increased accuracy in the determination of the temperature and coefficient of thermal expansion of the part or standard being measured is of increasing importance when state-of-the-art precision-tolerance parts are being inspected. Finally, the paper’s results support the view that a change in the reference temperature from 20 °C to 23 °C, without recalibration of reference standards at the new temperature, can introduce changes in dimensions and uncertainties in dimensional measurements which are substantial compared to manufacturing tolerances and industrial measurement-accuracy requirements.

## Figures and Tables

**Table 1:** Variety of values of coefficients of thermal expansion (in ppm/°C) of some metrologically-important materials provided in various engineering references

Material	CRC [11]	MHB [12]	MSG [13]	ASM [14]	TPM [16]
Al	25	22.4		23.6	23.1
Al 6061			22.0	23.4	22.5
SS 304	17.3		10.6–17.8*	17.2	14.7
BeCu	16.7				16.2
Fe	12			11.7	11.8
Cast iron	13.5	11.8	10.6–18.7	8.1–19.3	11.9
C-Steel	12.1	11.4	13.5–15.2	11.6–12.6	10.7
Pyrex	3.2			3.2	2.8
Silicon	3		4.67	5	2.6
Fused quartz	0.42		0.56	0.55	0.49
Invar				0.64–2.0	0.13
Zerodur [12]					0.05

* Source identifies stainless steels only by type, e.g. austenitic, ferritie, and age-hardenable.

**Table 2:** Variety of values of the coefficient of thermal expansion (CTE, in ppm/°C) of carbon steel reported in various sources

MHB [12]	CRC [11]	MSG [13]	ASM-1 [15]	ASM-2 [14]	TPM [10]
Steel, carbon	Plain carbon steel AISI-1020	Carbon steel hardening grades wrought *T* = 21 °C–649 °C 133–14.9	AISI grade 1020 (0.22%C) *T* = 20 °C–100°C 11.7	Fe-C alloy 1.08% C *T* = 20 °C–100°C 10.8	Carbon steel Fe + (0.7–1.4)%C well-annealed *T* = 20 °C 10.7 ±0.7
11.4	Typical 12.1	Carbon steel carburizing grades wrought *T* = 21 °C–649 °C 15.2	AISI grades 1070–1085 *T* = 20 °C–100°C 11.0–11.8	Fe-Calloys 1.45% C *T* = 20 °C–100 °C 10.1	

**Table 3:** Effects of heat treatment and mechanical processing on the mean thermal expansion of Invar (*T*=16 °C–100 °C)

Processing	Mean *α* (ppm/°C)
Quenched cold-drawn	0.14
Annealed quenched	0.5
Hoi mill	1.4
Forged	1.7
19 h–cool from 830 °C	2.0

**Table 4:** Calculated temperature-average (20°C–107°C) and temperature derivatives (20 °C) of thermal expansion coefficients (CTEs) for some metrologically important materials [11,12]

Material	*α*_av_ (20 °C–107 °C) (ppm/°C)	*α*(20 °C) (ppm/°C)	(d*α/*d*T*)_20_ *_°_*_C_ [ppm/(°C)]^2^	(d*α*/*α*d*T*) (%/°C)
Aluminum	24.2	23.1	0.009	0.04
Al 6061	23.7	22.5	0.023	0.10
BeCu		16.2	av 0.009_280–299_	0.06
Cast iron	12.0	11.9	0.0088	0.07
C-steel	11.9	10.7	0.018	0.17
Quartz	11.7	10.3	0.023	0.22
Pyrex	3.0	2.8	0.00083	0.03
Silicon	3.1	2.6	0.0031	0.12
Fused quartz	0.60	0.49	0.00032	0.07
Invar	0.56	0.13	0.012	9.2
Zerodur	0.05	<0.05	< 0.0015_293–318_	

**Table 5:** Comparison of the stated uncertainties in coefficients of thermal expansions associated with various standard gages, data, and materials

Specifier	Material	*α* (ppm/°C)	*Δ_α_/α* (%)	*Δ_α_* (ppm/°C)
ANSI standard for gage blocks [22]	Stainless steel	To be stated by manufacturer of G-blocks	± 10% of stated value	1–1.5
	Cr-plated steel			1.1
	Chrome carbide			0.8
	Tungsten carbide			0.4
TPM standard reference data [17]	Aluminum	23.1	3%	0.7
	Al 6061	22.5	7%	1.6
	Carbon steel	10.7	7%	0.75
	Silicon	2.6	5%	0.13
	Fused quartz	0.49	5%	0.025
NIST standard reference matls [24]	Copper	16.64	0.18%	0.03
	SS-446	9.76	0.31%	0.03
	BS-glass	4.78	0.63%	0.03
	Fused SiO_2_	0.48	6.3%	0.03
NRLM dilatometer results [25]	Duraluminum	23.129	0.37%	0.086
	Copper	16.556	0.33%	0.055
	C-steel (0.55%)	11.314	0.36%	0.038
	Invar	0.351	2.0%	0.007
	Glass ceramic	0.000		0.006

**Table 6:** Stated (*Δ_τ_*) and expanded (*U_τ_*) uncertainties in temperature measurement near 20 *°*C altainable by standard platinum resistance, bead-in-glass thermistor, type-T thermocouple, and mercury-in-glass thermometers

Sensor	Reference	Instrument	Balh	*Δ_τ_* (stated)	*U_τ_* (expanded)
SPRT	Ga-Pt			0.0001 °C (*σ*)	0.0002 °C
SPRT	SPRT	Bridge	20 °C Cell	0.001 °C (*σ*)	0.002 °C
TC	SPRT	Bridge	20 °C Cell	0.002 °C	0.0023 °C
Thermistor		Bridge		0.01 °C	0.012 °C
Hg-glass				0.03 °C	0.035 °C
TC		DVM	0 °C June	0.1 °C	0.12 °C

**Table 7:** Temperature stabilities and uncertainties reported for various state-of-the-art dimensional-measurement instruments and facilities

Instrument/facility with high-performance temperature system	Reported “stability”	Reported “accuracy”	Expanded uncertainty
Primary-std linescale calibration		0.002 °C	0.0023 °C
Large-optics-diamond-turning machine	0.006 °C	0.01 °C	0.010 °C
Primary-std-lab CMM calibration		0.01 °C	0.012 °C
Commercial IC mask metrology system	0.01 °C		0.012 °C
Commercial IC mask metrology system	0.05 °C		0.058 °C
Conventional CMM laboratory		0.1 °C	0.12 °C

**Table 8:** Stated incremental, fractional length and fractional tolerance uncertainties compared to the Thermal Error Indices (*TEI*) for three state-of-the-art engineering measurement systems

	Rocket motor seal	CMM step gage	X-ray mask
Dimension	3650 mm	1000 mm	50 mm
Materials	Aluminum/steel	Steel/Zerodur	Silicon
*α* (ppm/°C)	23.4/12.2	11.5/0.00	2.8
*Δ_α_* (ppm/°C)	1.2/0.6 (5%)	0.1/0.05	(3%)
(*T−T*_0_)	Worst: 11.1 °C	1 °C	0 °C
	Ideal: 0 °C		
*Δ_τ_*	Worst: 0.9 °C	0.1 °C	0.01 °C
	Ideal: 0.36 °C		
*τ*	127 µm	1.33 µm	1.5 nm
*Δ*_L_	Worst: 95.3 µm	Steel: 1.80/1.27 µm	1 nm
	Ideal: 17.6 µm	Z-dur: 0.61/0.55 µm	
*Δ*_L_/*L*	Worst: 27 ppm	Steel: 1.8/1.3 ppm	0.02 ppm
	Ideal: 4.8 ppm	Z-dur: 0.6/0.6 ppm	
*Δ*_L_ */τ*	Worst: 75%	Steel: 135%/96%	67%
	Ideal: 14%	Z-dur: 46%/41%	
*TEI*	Worst: 47%	Steel: 94%	67%
	Ideal: 12%	Z-dur: 4%	

**Table 9:** Comparison of Thermal error indices (*TEI*), based on stated uncertainties, and Thermal Uncertainty Indices (*TUI*), based on expanded uncertainties, for various situation and thermal conditions

	ITS-90	Lab 20°C	MC goal	Primary	Secondary	Tertiary	L-screw	Piston
Dimension	1 m	1 m	70 mm	1 m	1 m	1 m	1000 mm	100 mm
Material	Si-to-Al	Si-to-Al	Si	Steel	Steel	Steel	Steel	Al
*α* (ppm/°C)	2.5–25	2.5–25	2.6	11.75	11.8	11.8	11.5	23.4
(*T−T*_0_)			0.000°C	0.01 °C	0.1 °C	1.0	3 °C	3 °C
*Δ_α_* (ppm/°C)				0.03	0.03	0.6	⩾0.6	
*Δ_T_*	0.0001 °C^a^	0.001 °C	0.001 °C	0.002 °C	0.01 °C	0.1		
*τ*			1 nm	0.1 μm	1.25 μm	12 μm	33.3 μm/m	7.6 μm
*Δ_L_*	0.25–2.5 nm	2.5–25 nm	0.18 nm	24 nm	0.12 μm	1.8 μm	1.8 μm/m	7.0 μm
*Δ_L_/l*	⩾2.5·10^−10^	⩾2.5·10^−9^	2.6·10^−9^	2.4·10^−8^	1·10^−7^	2·10^−6^	1.8·10^−6^	7·10^−5^
*TEI*			18%	25%	10%	24%	5%	92%
*U_α_* (ppm/°C)					0.035	0.035	0.7	0.7
*U_T_*	0.0002 °C	0.0012 °C	0.0012 °C	0.0023 °C	0.012 °C	0.12 °C		
*τ*			1.2 nm	0.12 μm	1.4 μm	13.8 μm	38.3 μm	8.7 μm
*U_L_*	0.5–5 nm	2.9–29 nm	0.22 nm	27.6 nm	0.14 μm	1.3 nm	2.1 μm/m	7.0 μm
*U_L_*/*L*	⩾5·10^−10^	⩾3·10^−9^	3.1·10^−9^	2.8·10^−8^	1.4·10^−7^	1.3·10^−6^	2.1·10^−6^	7·10^−5^
*TUI*			18%	23%	14%	10%	5%	80%

a Stated uncertainty for this case was one standard deviation; all other examples were unspecified and treated as uniform distributions.

**Table 10:** Relative expanded uncertainty in length for low-expansion materials due to the 0.00O2 °C limit of ITS-90 compared to the relative expanded uncertainty in length due to uncertainty in the I-HeNe wavelength

Matl	*α*(ppm/°C)	*U_L_*/*L*
Steel	11.5	23.0×10^−10^
Silicon	2.6	5.2×10^−10^
Invar	1.0	2.0×10^−10^
Fused quartz	0.4	0.8×10^−10^
^127^I_2_-HeNe	0.25	0.5×10^−10^
Zerodur	0.05	0.1×10^−10^

## Footnotes

1 Certain commercial equipment, instruments, or materials are identified in this paper to specify adequately the experimental procedure. Such identification does not imply recommendation or endorsement by the National Institute of Standards and Technology, nor does it imply that the materials or equipment identified are necessarily the best available for the purpose.
